# Penta­carbonyl-1κ^2^ *C*,2κ^3^ *C*-(4-iodo­phenyl isocyanide-1κ*C*)(μ-propane-1,3-dithiol­ato-1:2κ^4^ *S*,*S*′:*S*,*S*′)iron(I)(*Fe*—*Fe*)

**DOI:** 10.1107/S1600536811047088

**Published:** 2011-11-12

**Authors:** Jinli Zhu, Yanfeng Tang, Guo -Min Jiang, Miao Wang, Ping Hua

**Affiliations:** a College of Chemistry and Chemical Engineering, Nantong University, Nantong 226019, People’s Republic of China

## Abstract

In the title compound, [Fe_2_(C_7_H_4_IN)(C_3_H_6_S_2_)(CO)_5_], the Fe—Fe distance of 2.5156 (11) Å compares well with that in related model structures. The phenyl isocyanide ligand is in the basal position and *trans* to the S atoms of the propane­dithiol­ate ligand due to steric hindrance. The crystal structure features C—H⋯O inter­actions.

## Related literature

The title compound was prepared as a model for the iron-only hydrogenase ([Fe]H_2_ase) active site. Iron-only hydrogenase in micro-organisms can catalyse the reversible reduction of protons to hydrogen, see: Cammack (1999 ▶); Frey (2002 ▶); Nicolet *et al.* (2000 ▶). For the active site of [Fe]H_2_ase, see: Nicolet *et al.* (1999 ▶); Peters *et al.* (1998 ▶). For an analogous structure, see: Lyon *et al.* (1999 ▶). For the preparation of the starting material [Fe_2_(S_2_C_3_H_6_)(CO)_6_], see: Winter *et al.* (1982 ▶). 
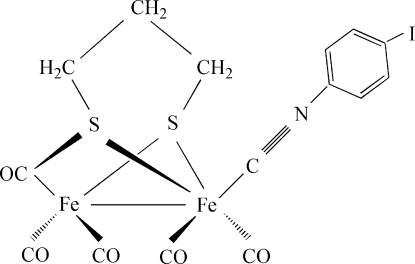


## Experimental

### 

#### Crystal data

[Fe_2_(C_7_H_4_IN)(C_3_H_6_S_2_)(CO)_5_]
*M* *_r_* = 586.96
Monoclinic, 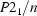

*a* = 7.7290 (3) Å
*b* = 11.7215 (5) Å
*c* = 22.3974 (10) Å
β = 99.466 (1)°
*V* = 2001.47 (15) Å^3^
*Z* = 4
Mo *K*α radiation
μ = 3.23 mm^−1^
*T* = 293 K
0.15 × 0.14 × 0.12 mm

#### Data collection

Bruker Smart APEX CCD area-detector diffractometer
Absorption correction: multi-scan (*SADABS*; Bruker, 2000 ▶) *T*_min_ = 0.630, *T*_max_ = 0.680
5430 measured reflections
3254 independent reflections
2913 reflections with *I* > 2σ(*I*)
*R*_int_ = 0.018

#### Refinement

*R*[*F* ^2^ > 2σ(*F* ^2^)] = 0.051
*wR*(*F* ^2^) = 0.110
*S* = 1.09
3254 reflections
235 parameters
219 restraints
H-atom parameters constrained
Δρ_max_ = 0.78 e Å^−3^
Δρ_min_ = −0.64 e Å^−3^

### 

Data collection: *SMART* (Bruker, 2000 ▶); cell refinement: *SAINT* (Bruker, 2000 ▶); data reduction: *SAINT*; program(s) used to solve structure: *SHELXS97* (Sheldrick, 2008 ▶); program(s) used to refine structure: *SHELXL97* (Sheldrick, 2008 ▶); molecular graphics: *SHELXTL* (Sheldrick, 2008 ▶); software used to prepare material for publication: *SHELXTL*.

## Supplementary Material

Crystal structure: contains datablock(s) global, I. DOI: 10.1107/S1600536811047088/zj2032sup1.cif

Structure factors: contains datablock(s) I. DOI: 10.1107/S1600536811047088/zj2032Isup2.hkl

Additional supplementary materials:  crystallographic information; 3D view; checkCIF report

## Figures and Tables

**Table 1:** Selected bond lengths (Å)

Fe1—C2	1.785 (7)
Fe1—C1	1.805 (6)
Fe1—C3	1.812 (6)
Fe1—S1	2.2656 (17)
Fe1—S2	2.2692 (15)
S1—Fe2	2.2590 (16)
Fe2—C8	1.785 (6)
Fe2—C7	1.797 (6)
Fe2—C9	1.867 (6)
Fe2—S2	2.2689 (14)

**Table 2:** Hydrogen-bond geometry (Å, °)

*D*—H⋯*A*	*D*—H	H⋯*A*	*D*⋯*A*	*D*—H⋯*A*
C12—H12*A*⋯O2^i^	0.93	2.57	3.423 (9)	153
C15—H15*A*⋯O5^ii^	0.93	2.55	3.464 (8)	168

Symmetry codes: (i) 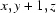; (ii) 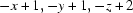.

**Fig. 1.**
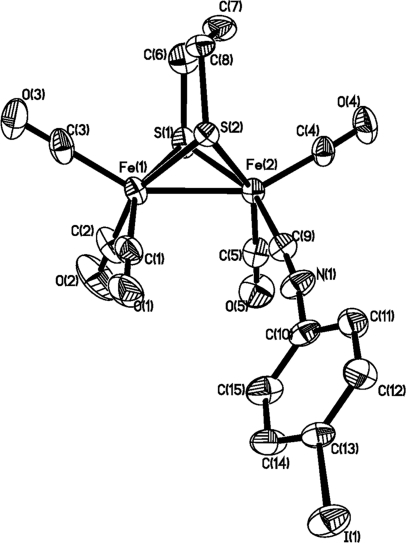
View of the title compound, with the atomic numbering scheme. Displacement ellipsoids are drawn at the 50% probability level.

**Fig. 2.**
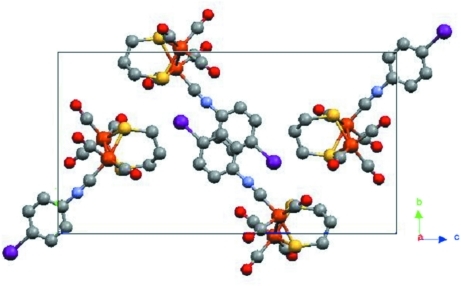
A packing diagram for the title compound viewed along the [001] axis (H atoms are omitted for clarity)

## supplementary crystallographic information

### Comment

The iron-only Hydrogenases ([Fe]H_2_ase) in microorganisms can catalyze the reversible reduction of protons to hydrogen according to the reaction: 2H^+^+2 e^-^=H_2_. (Cammack *et al.* 1999, Nicolet *et al.* 2000, Frey *et al.* 2002) The active site of [Fe]H_2_ase is consisted of a 2Fe_2_S linked to a [4Fe_4_S] cluster by a bridged cysteine sulfur (Peters *et al.* 1998, Nicolet *et al.* 1999). In the 2Fe_2_S unit, the two iron atoms are coordinated by CO and CN– ligands. We have prepared the title complex as a structural model for the iron-only hydrogenases active site. Herein we report its crystal structure.

The molecular structure of the title complex is shown in Fig.1 and selected bond distances are listed in Table 1. The crystal packing diagram reveals that molecules of the title compound form layers in the yz plane (Fig. 2) and the intermolecular interactions present in the structure are listed in Table 2. The Fe—Fe distance of 2.5157 (14) Å compares well with that in the (µ-PDT) Fe_2_ (CO)_6_ analogous structure (Lyon *et al.*, 1999). The phenyl isocyanide ligand is in the basal position and *trans* to the sulfur atoms of the propanedithiolate ligand due to the steric hindrance. The Fe—CN distance of 1.87 Å is longer than Fe—CO distance of 1.79–1.81 Å, suggesting the strong electron σ-donating of the isocyanide ligand with iron center. The π-π conjugation between CN triple bond and phenyl ring is somewhat interrupted in the solid state, as the angle of C(9) N(1) C(10) is 169.8 (9)°, indicating a slight distorting from linearity.

### Experimental

A solution of Fe_2_(S_2_C_3_H_6_)(CO)_6_ (Winter *et al.* 1982) (1.5 g, 3.88 mmol) in 100 ml MeCN was treated with a solution of Me_3_NO.2H_2_O (433 mg, 3.9 mmol) in 30 ml of MeCN followed by a solution of *p*- benzylisocyanide (895 mg, 3.9 mmol) in 30 ml of MeCN at ambient temperature. After 2 h at this temperature, the solvent was removed in vacuo, and the resulting red residue was purified on silica gel to give title compound as a red solid (1.94 g, 85% yield). Single crystals of the title compound for X-ray analysis were grown by slow evaporation. A near saturated solution of the title compound was prepared in a CH_2_Cl_2_ -hexane (1:5 *v*/*v*) solution. The solution was then being left in a glass tube that has a perforated cap at ambient temperature. After one week, some of the crystals grow on the side of the tube.

### Refinement

Carbon-bound H atoms were positioned geometrically, with C—H = 0.97Å for methylene and 0.93 Å for aromatic, and refined using a riding model, with *U*_iso_ (H) = 1.2 *U*_eq_ (C). The hydroxyl H atom was positioned geometrically and freely refined.

Data collection: *SMART* (Bruker, 2000); cell refinement: *SAINT* (Bruker, 2000); data reduction: *SAINT*; program(*s*) used to solve structure: *SHELXTL* (Sheldrick, 2008); program(*s*) used to refine structure: *SHELXTL*; molecular graphics: *SHELXTL*; software used to prepare material for publication: *SHELXTL*.

### Crystal data

**Table:**

[Fe_2_(C_7_H_4_IN)(C_3_H_6_S_2_)(CO)_5_]	*Z* = 4
*M**_r_* = 586.96	*F*(000) = 1136
Monoclinic, *P*2_1_/*n*	*D*_x_ = 1.948 Mg m^−^^3^
Hall symbol: -P 2yn	Mo *K*α radiation, λ = 0.71073 Å
*a* = 7.7290 (3) Å	µ = 3.23 mm^−^^1^
*b* = 11.7215 (5) Å	*T* = 293 K
*c* = 22.3974 (10) Å	Cuboid, red
β = 99.466 (1)°	0.15 × 0.14 × 0.12 mm
*V* = 2001.47 (15) Å^3^	

### Data collection

**Table:**

Bruker Smart APEX CCD area-detector diffractometer	3254 independent reflections
Radiation source: fine-focus sealed tube	2913 reflections with *I* > 2σ(*I*)
graphite	*R*_int_ = 0.018
phi and ω scans	θ_max_ = 24.5°, θ_min_ = 1.8°
Absorption correction: multi-scan (*SADABS*; Bruker, 2000)	*h* = −6→9
*T*_min_ = 0.630, *T*_max_ = 0.680	*k* = −13→10
5430 measured reflections	*l* = −22→25

### Refinement

**Table:**

Refinement on *F*^2^	Primary atom site location: structure-invariant direct methods
Least-squares matrix: full	Secondary atom site location: difference Fourier map
*R*[*F*^2^ > 2σ(*F*^2^)] = 0.051	Hydrogen site location: inferred from neighbouring sites
*wR*(*F*^2^) = 0.110	H-atom parameters constrained
*S* = 1.09	*w* = 1/[σ^2^(*F*_o_^2^) + (0.0001*P*)^2^ + 16.8715*P*] where *P* = (*F*_o_^2^ + 2*F*_c_^2^)/3
3254 reflections	(Δ/σ)_max_ = 0.002
235 parameters	Δρ_max_ = 0.78 e Å^−^^3^
219 restraints	Δρ_min_ = −0.64 e Å^−^^3^

### Special details

**Table:**

Geometry. All e.s.d.'s (except the e.s.d. in the dihedral angle between two l.s. planes) are estimated using the full covariance matrix. The cell e.s.d.'s are taken into account individually in the estimation of e.s.d.'s in distances, angles and torsion angles; correlations between e.s.d.'s in cell parameters are only used when they are defined by crystal symmetry. An approximate (isotropic) treatment of cell e.s.d.'s is used for estimating e.s.d.'s involving l.s. planes.
Refinement. Refinement of *F*^2^ against ALL reflections. The weighted *R*-factor *wR* and goodness of fit *S* are based on *F*^2^, conventional *R*-factors *R* are based on *F*, with *F* set to zero for negative *F*^2^. The threshold expression of *F*^2^ > σ(*F*^2^) is used only for calculating *R*-factors(gt) *etc*. and is not relevant to the choice of reflections for refinement. *R*-factors based on *F*^2^ are statistically about twice as large as those based on *F*, and *R*- factors based on ALL data will be even larger.

### Fractional atomic coordinates and isotropic or equivalent isotropic displacement parameters (Å^2^)

**Table:**

	*x*	*y*	*z*	*U*_iso_*/*U*_eq_	
N1	0.3432 (6)	0.7795 (5)	0.9391 (2)	0.0648 (15)	
I1	0.13693 (6)	1.11048 (4)	1.135787 (19)	0.07267 (14)	
Fe1	0.09353 (10)	0.48170 (7)	0.86223 (4)	0.0473 (2)	
S1	0.28437 (19)	0.42518 (11)	0.80138 (7)	0.0531 (4)	
O1	−0.0108 (7)	0.6147 (5)	0.9623 (2)	0.0910 (16)	
C1	0.0272 (8)	0.5622 (5)	0.9234 (3)	0.0599 (14)	
Fe2	0.36277 (9)	0.59245 (6)	0.84823 (3)	0.04142 (18)	
S2	0.08546 (16)	0.64796 (11)	0.80994 (6)	0.0403 (3)	
O2	0.2466 (8)	0.2970 (5)	0.9430 (3)	0.124 (2)	
C2	0.1834 (9)	0.3696 (6)	0.9117 (3)	0.0699 (14)	
O3	−0.2423 (6)	0.3742 (5)	0.8099 (2)	0.0927 (17)	
C3	−0.1125 (8)	0.4154 (5)	0.8299 (3)	0.0605 (13)	
O4	0.6000 (6)	0.6782 (4)	0.7683 (2)	0.0833 (14)	
C4	0.1905 (9)	0.4428 (5)	0.7209 (3)	0.0649 (14)	
H4A	0.2723	0.4100	0.6972	0.078*	
H4B	0.0839	0.3978	0.7129	0.078*	
O5	0.6200 (6)	0.4745 (5)	0.9381 (2)	0.0909 (17)	
C5	0.1472 (9)	0.5607 (6)	0.6976 (3)	0.0666 (15)	
H5A	0.2554	0.6040	0.7012	0.080*	
H5B	0.0990	0.5555	0.6549	0.080*	
C6	0.0215 (8)	0.6253 (5)	0.7284 (2)	0.0564 (14)	
H6A	−0.0898	0.5852	0.7217	0.068*	
H6B	0.0024	0.6993	0.7091	0.068*	
C7	0.5080 (8)	0.6483 (5)	0.8004 (3)	0.0553 (13)	
C8	0.5211 (8)	0.5203 (5)	0.9022 (3)	0.0580 (14)	
C9	0.3564 (7)	0.7105 (5)	0.9038 (3)	0.0524 (12)	
C10	0.3002 (8)	0.8540 (5)	0.9841 (2)	0.0542 (12)	
C11	0.2862 (8)	0.9692 (5)	0.9732 (3)	0.0573 (12)	
H11A	0.3087	0.9986	0.9366	0.069*	
C12	0.2389 (8)	1.0414 (5)	1.0164 (3)	0.0576 (13)	
H12A	0.2269	1.1193	1.0087	0.069*	
C13	0.2097 (7)	0.9983 (5)	1.0706 (2)	0.0512 (12)	
C14	0.2235 (9)	0.8808 (5)	1.0821 (3)	0.0616 (13)	
C15	0.2705 (8)	0.8104 (5)	1.0382 (3)	0.0614 (13)	
H15A	0.2822	0.7324	1.0455	0.074*	
H14A	0.1991	0.8516	1.1182	0.074*	

### Atomic displacement parameters (Å^2^)

**Table:**

	*U*^11^	*U*^22^	*U*^33^	*U*^12^	*U*^13^	*U*^23^
N1	0.057 (3)	0.073 (3)	0.062 (3)	0.007 (3)	0.004 (2)	−0.029 (3)
I1	0.0759 (3)	0.0804 (3)	0.0617 (2)	0.0098 (2)	0.0113 (2)	−0.0295 (2)
Fe1	0.0490 (4)	0.0437 (4)	0.0540 (4)	−0.0003 (3)	0.0224 (3)	0.0047 (3)
S1	0.0550 (7)	0.0342 (7)	0.0756 (9)	0.0039 (6)	0.0268 (7)	−0.0082 (6)
O1	0.118 (3)	0.100 (4)	0.067 (3)	−0.016 (3)	0.051 (2)	−0.017 (3)
C1	0.058 (3)	0.064 (3)	0.062 (3)	0.002 (2)	0.024 (2)	0.004 (2)
Fe2	0.0412 (4)	0.0367 (4)	0.0491 (4)	0.0025 (3)	0.0154 (3)	−0.0058 (3)
S2	0.0453 (6)	0.0367 (6)	0.0408 (6)	0.0046 (5)	0.0126 (5)	−0.0034 (5)
O2	0.130 (4)	0.111 (4)	0.138 (4)	0.038 (3)	0.048 (3)	0.072 (3)
C2	0.071 (3)	0.065 (3)	0.078 (3)	0.007 (2)	0.026 (2)	0.019 (2)
O3	0.071 (3)	0.095 (4)	0.117 (4)	−0.030 (3)	0.033 (3)	−0.032 (3)
C3	0.061 (2)	0.058 (3)	0.069 (3)	−0.005 (2)	0.029 (2)	−0.007 (2)
O4	0.082 (3)	0.079 (3)	0.100 (3)	−0.022 (3)	0.050 (2)	−0.004 (3)
C4	0.075 (3)	0.063 (3)	0.061 (3)	−0.002 (2)	0.023 (2)	−0.020 (2)
O5	0.077 (3)	0.089 (3)	0.101 (4)	0.022 (3)	−0.002 (3)	0.013 (3)
C5	0.074 (3)	0.075 (3)	0.052 (3)	−0.002 (3)	0.016 (2)	−0.012 (3)
C6	0.064 (3)	0.061 (3)	0.045 (2)	−0.004 (3)	0.010 (2)	−0.008 (2)
C7	0.059 (2)	0.050 (2)	0.062 (2)	−0.005 (2)	0.022 (2)	−0.011 (2)
C8	0.055 (3)	0.052 (3)	0.069 (3)	0.006 (2)	0.018 (2)	−0.005 (2)
C9	0.054 (2)	0.051 (2)	0.054 (2)	0.006 (2)	0.013 (2)	−0.007 (2)
C10	0.062 (2)	0.053 (2)	0.046 (2)	0.006 (2)	0.006 (2)	−0.013 (2)
C11	0.071 (2)	0.053 (2)	0.049 (2)	0.000 (2)	0.013 (2)	−0.007 (2)
C12	0.073 (3)	0.049 (2)	0.050 (2)	0.005 (2)	0.010 (2)	−0.004 (2)
C13	0.061 (2)	0.050 (2)	0.041 (2)	0.008 (2)	0.005 (2)	−0.009 (2)
C14	0.081 (3)	0.055 (2)	0.047 (2)	0.004 (2)	0.004 (2)	−0.004 (2)
C15	0.078 (2)	0.049 (2)	0.055 (2)	0.007 (2)	0.002 (2)	−0.007 (2)

### Geometric parameters (Å, °)

**Table:**

N1—C9	1.148 (7)	C4—C5	1.495 (9)
N1—C10	1.413 (7)	C4—H4A	0.9700
I1—C13	2.109 (5)	C4—H4B	0.9700
Fe1—C2	1.785 (7)	O5—C8	1.148 (7)
Fe1—C1	1.805 (6)	C5—C6	1.488 (9)
Fe1—C3	1.812 (6)	C5—H5A	0.9700
Fe1—S1	2.2656 (17)	C5—H5B	0.9700
Fe1—S2	2.2692 (15)	C6—H6A	0.9700
Fe1—Fe2	2.5156 (11)	C6—H6B	0.9700
S1—C4	1.841 (6)	C10—C15	1.370 (8)
S1—Fe2	2.2590 (16)	C10—C11	1.373 (8)
O1—C1	1.144 (7)	C11—C12	1.380 (8)
Fe2—C8	1.785 (6)	C11—H11A	0.9300
Fe2—C7	1.797 (6)	C12—C13	1.369 (8)
Fe2—C9	1.867 (6)	C12—H12A	0.9300
Fe2—S2	2.2689 (14)	C13—C14	1.401 (8)
S2—C6	1.831 (5)	C14—C15	1.376 (8)
O2—C2	1.157 (8)	C14—H14A	0.9272
O3—C3	1.137 (7)	C15—H15A	0.9300
O4—C7	1.146 (7)		
			
C9—N1—C10	170.0 (6)	C5—C4—S1	118.4 (4)
C2—Fe1—C1	92.5 (3)	C5—C4—H4A	107.7
C2—Fe1—C3	99.2 (3)	S1—C4—H4A	107.7
C1—Fe1—C3	100.8 (3)	C5—C4—H4B	107.7
C2—Fe1—S1	86.2 (2)	S1—C4—H4B	107.7
C1—Fe1—S1	154.6 (2)	H4A—C4—H4B	107.1
C3—Fe1—S1	104.5 (2)	C6—C5—C4	115.8 (6)
C2—Fe1—S2	158.1 (2)	C6—C5—H5A	108.3
C1—Fe1—S2	87.5 (2)	C4—C5—H5A	108.3
C3—Fe1—S2	102.4 (2)	C6—C5—H5B	108.3
S1—Fe1—S2	84.61 (5)	C4—C5—H5B	108.3
C2—Fe1—Fe2	102.2 (2)	H5A—C5—H5B	107.4
C1—Fe1—Fe2	99.7 (2)	C5—C6—S2	116.5 (4)
C3—Fe1—Fe2	149.5 (2)	C5—C6—H6A	108.2
S1—Fe1—Fe2	56.10 (4)	S2—C6—H6A	108.2
S2—Fe1—Fe2	56.33 (4)	C5—C6—H6B	108.2
C4—S1—Fe2	112.9 (2)	S2—C6—H6B	108.2
C4—S1—Fe1	111.4 (2)	H6A—C6—H6B	107.3
Fe2—S1—Fe1	67.56 (5)	O4—C7—Fe2	176.2 (5)
O1—C1—Fe1	178.2 (6)	O5—C8—Fe2	178.0 (6)
C8—Fe2—C7	98.6 (3)	N1—C9—Fe2	175.5 (6)
C8—Fe2—C9	89.5 (3)	C15—C10—C11	120.3 (5)
C7—Fe2—C9	102.4 (3)	C15—C10—N1	119.6 (5)
C8—Fe2—S1	90.3 (2)	C11—C10—N1	120.1 (5)
C7—Fe2—S1	100.69 (19)	C10—C11—C12	120.0 (6)
C9—Fe2—S1	156.70 (19)	C10—C11—H11A	120.0
C8—Fe2—S2	153.1 (2)	C12—C11—H11A	120.0
C7—Fe2—S2	108.30 (19)	C13—C12—C11	119.8 (6)
C9—Fe2—S2	84.98 (17)	C13—C12—H12A	120.1
S1—Fe2—S2	84.77 (5)	C11—C12—H12A	120.1
C8—Fe2—Fe1	99.2 (2)	C12—C13—C14	120.5 (5)
C7—Fe2—Fe1	150.77 (19)	C12—C13—I1	119.0 (4)
C9—Fe2—Fe1	100.75 (18)	C14—C13—I1	120.5 (4)
S1—Fe2—Fe1	56.35 (5)	C15—C14—C13	118.7 (6)
S2—Fe2—Fe1	56.34 (4)	C15—C14—H14A	121.2
C6—S2—Fe1	111.7 (2)	C13—C14—H14A	120.1
C6—S2—Fe2	114.7 (2)	C10—C15—C14	120.7 (6)
Fe1—S2—Fe2	67.33 (4)	C10—C15—H15A	119.7
O2—C2—Fe1	177.7 (7)	C14—C15—H15A	119.7
O3—C3—Fe1	179.5 (6)		

### Hydrogen-bond geometry (Å, °)

**Table:**

*D*—H···*A*	*D*—H	H···*A*	*D*···*A*	*D*—H···*A*
C12—H12A···O2^i^	0.93	2.57	3.423 (9)	153
C15—H15A···O5^ii^	0.93	2.55	3.464 (8)	168

Symmetry codes: (i) *x*, *y*+1, *z*; (ii) −*x*+1, −*y*+1, −*z*+2.
